# In This Issue

**DOI:** 10.1002/cfg.501

**Published:** 2005


**Comparative and Functional Genomics**
*Comp Funct Genom* 2005; **6**: 343.
Published online in Wiley InterScience (www.interscience.wiley.com). **DOI:** 10.1002/cfg.501

### In this issue

### Editorial — Goodbye to CFG

Sadly, this will be the last issue of *Comparative and Functional Genomics*. Our Editor-in-Chief, **Stephen Oliver**, thanks all those who were involved in publishing the journal.

### *In silico* analysis for transcription factors with $Zn(II)_2C_6$ binuclear cluster DNA binding domains in *Candida albicans*

6047 *C. albicans* open reading frames were screened for $Zn(II)_2C_6$-type zinc cluster domains (or binuclear cluster domains) involved in DNA recognition, by **Sergi Maicas** *et al.* These fungal transcription regulators control genes involved in a wide range of cellular processes including metabolism of different compounds such as sugars or amino acids, multi-drug resistance, control of meiosis, cell wall architecture, etc. The selection criteria used in the sequence analysis were the presence of the $CysX_2CysX_6CysX_{5-16}CysX_2CysX_{6-8}$ Cys motif and a putative nuclear localization signal. Using this approach, 70 putative $Zn(II)_2C_6$ transcription factors were found in the genome of *C. albicans*.

### Meeting Review — eGenomics: genomes and the environment

Environmental genomics is the investigation of how living organisms adapt to and are impacted by their environments using genomic technologies. **Dawn Field and colleagues** report from a workshop held on September 5$^{th}$ to 6$^{th}$ 2005, which aimed to examine current work at the interface between genomes and environmental science and to explore future prospects for the development of this important interface.

### Meeting Review — eGenomics: Cataloguing our Complete Genome Collection

There is increasing recognition that the scientific community would benefit from the development of a new standard to capture a richer set of information describing complete genomes. **Dawn Field and colleagues** report from a Workshop held on September 7$^{th}$ to 9$^{th}$ 2005 with the aim of addressing this need.

### Special section of papers from the Bio-Ontologies special interest group meeting at ISMB 2005

**Robert Stevens and co-organisers** open this special section with a report from the 8$^{th}$ Annual Bio-Ontologies special interest group meeting.

**Mary Shimoyama and colleagues** describe how the Rat Genome Database has developed a comprehensive ontology-based data structure and annotation system to integrate physiological data with environmental and experimental factors, and genetic and genomic information.

**Barry Smith** *et al.* present an extension of their approach (in which the provision of formal definitions for relations such as *is_a, part_of* and *transformation_of* can facilitate the integration of biomedical ontologies) to the treatment of pathologies, focusing especially on those pathological continuant entities that arise when organs become affected by carcinomas.

**Pankaj Jaiswal and colleagues** describe the Plant Ontology, a collaborative effort among several plant databases and experts in plant systematics, botany and genomics, and give us a detailed update on the current version, which integrates diverse vocabularies used to describe anatomy, morphology and growth stages of *Arabidopsis*, maize and rice.

**Daniel McShan** discusses the state of the art in biochemical databases and how these sources may be applied to the problem of classifying compounds based solely on structure. He describes a biochemical informatics system for processing molecular data and describes how 100,255 compositional (hasA) relationships are inferred between 835 abstractions and 9500 metabolites from the KEGG Ligand database.

### Conference calendar

A listing of genomics related conferences planned for March to May 2006.

### Thank you to reviewers

We take the opportunity to thank all our external reviewers of papers for CFG.

Copyright © 2006 John Wiley & Sons, Ltd.

